# Prognostic factors for relapse-free survival in stage IB-IIIA primary lung adenocarcinoma by epidermal growth factor receptor mutation status

**DOI:** 10.1186/s12885-022-10057-w

**Published:** 2022-09-09

**Authors:** Tetsuya Isaka, Hiroyuki Ito, Tomoyuki Yokose, Haruhiro Saito, Hiroyuki Adachi, Kotaro Murakami, Jun Miura, Noritake Kikunishi, Yasushi Rino

**Affiliations:** 1 Department of Thoracic Surgery, Kanagawa Cancer Center, 2-3-2 Nakao, Asahi, Yokohama, Kanagawa 241-8515 Japan; 2 Department of Surgery, Yokohama City University, 3-9 Fukuura, Kanazawa, Yokohama, Kanagawa 236-0004 Japan; 3 Department of Pathology, Kanagawa Cancer Center, 2-3-2 Nakao, Asahi, Yokohama, Kanagawa 241-8515 Japan; 4 Department of Thoracic Oncology, Kanagawa Cancer Center, 2-3-2 Nakao, Asahi, Yokohama, Kanagawa 241-8515 Japan

**Keywords:** EGFR mutation, Pathological stage, Relapse-free survival, Primary lung adenocarcinoma, Adjuvant chemotherapy

## Abstract

**Background:**

Pathological stage IB-IIIA lung adenocarcinoma with an epidermal growth factor receptor (EGFR) mutation (Mt) has a high recurrence rate even after complete resection. However, there have been few reports on the risk factors for Mt recurrence. This study aimed to analyze the clinicopathological factors related to the relapse-free survival (RFS) of patients with pathological stage IB-IIIA primary lung adenocarcinoma with and without an EGFR mutation.

**Methods:**

Patients who underwent curative surgery for Mt (*n* = 208) harboring the EGFR exon 21 L858R point mutation or EGFR exon 19 deletion mutation and EGFR mutation wild-type lung adenocarcinoma (Wt, *n* = 358) between January 2010 and December 2020 were included. Patients who received adjuvant EGFR-tyrosine kinase inhibitors were excluded. The prognostic factors for RFS were analyzed using a multivariable Cox regression analysis.

**Results:**

The 5-year RFS rates in the Mt and Wt groups were 43.5 and 52.3%, respectively (*p* = 0.907). Prognostic factors for RFS in the Mt group included smoking history (hazard ratio [HR], 1.49; *p* = 0.049), blood vessel invasion (HR, 1.84; *p* = 0.023), and lymph node metastasis (HR, 1.96; *p* = 0.005). However, adjuvant chemotherapy was not a prognostic factor (HR, 1.02; *p* = 0.906). In contrast, positron emission tomography (PET) max standardized uptake value (SUV) ≥ 6.0 (HR, 1.53; *p* = 0.042), lymphatic vessel invasion (HR, 1.54; *p* = 0.036), lymph node metastasis (HR, 1.79; *p* = 0.002), and adjuvant chemotherapy (HR, 0.60; *p* = 0.008) were prognostic factors for RFS in the Wt group.

**Conclusions:**

Prognostic factors for RFS in stage IB-IIIA primary lung adenocarcinoma differ by epidermal growth factor receptor mutation status. The impact of adjuvant chemotherapy on RFS also differed by EGFR mutation status.

## Background

Epidermal growth factor receptor (EGFR) mutations are found in 15–50% of non-small cell lung cancers (NSCLC), especially in women, non-smokers, Asians, and patients with adenocarcinoma [1, 2]. EGFR exon 19 deletion (Ex19) mutations and EGFR exon 21 L858R point mutation (Ex21) mutations are two major EGFR mutations and molecular targeted therapies, and EGFR-tyrosine kinase inhibitors (TKI) show antitumor effects against these cancers [3]. The overall survival (OS) of patients with unresectable EGFR mutation lung adenocarcinoma (Mt) has dramatically improved to 30–50 months with EGFR-TKI [4–6].

The postoperative recurrence rate of primary lung cancer has been reported to be 33% in pathological stage IB, 51% in stage II, and 61% in stage III [7]. Furthermore, the 5-year recurrence-free survival (RFS) rate for patients with Mt was poor (57.0% for stage IB, 46.6% for stage II, and 17.4% for stage IIIA) [8, 9]. To improve the postoperative prognosis of Mt, several clinical trials of adjuvant EGFR-TKI for pathological stage IB(II)-IIIA Mt have recently been conducted [10–14]. Adjuvant EGFR-TKI did not prolong RFS in the IMPACT trial [10]; however, the RADIENT, ADJUVANT, and ADAURA trials showed prolonged RFS with postoperative EGFR-TKI [11, 12, 14]. However, no clinical trials have shown that adjuvant EGFR-TKI prolongs overall survival (OS). The benefit of adjuvant EGFR-TKI is to improve RFS and reduce distant metastatic recurrence, especially brain and bone metastases, which leads to poor quality of life and poor prognosis [14–16]. Adjuvant EGFR-TKI in patients with favorable RFS would only increase drug toxicity [10, 12, 14] and would not be beneficial to the patient. Therefore, it is important to accurately assess the prognostic factors related to RFS.

The prognostic factors for RFS after complete resection of primary lung adenocarcinoma based on EGFR mutation status remain unclear. The aim of this study was to analyze clinicopathological prognostic factors for RFS after complete resection for pathologic stage IB-IIIA primary lung adenocarcinoma according to EGFR mutation status and to obtain basic knowledge on the selection of patients who should receive adjuvant therapy.

## Methods

### Patients and word definitions

This study was approved by the Kanagawa Cancer Center IRB (2021 Eki-153). Of 1081 cases of pathological stage IB-IIIA (8th edition TNM classification) NSCLC who underwent segmentectomy or greater extent of lung resection at Kanagawa Cancer Center between January 2010 and December 2020, 703 patients had primary lung adenocarcinoma without adjuvant EGFR-TKI therapy. Of these, 566 patients were included in the study, excluding 57 patients (8.1%) who did not undergo EGFR mutation examination, 20 patients with uncommon EGFR mutations (2.8%), such as EGFR exon 18 G719X, and 60 patients (8.5%) who did not undergo positron emission tomography (PET)-computed tomography (CT). In this study, 208 patients with Mt (*n* = 208) harboring Ex21 and Ex19 and 358 patients with wild-type EGFR mutations (Wt) were included. TNM staging was based on the 8th edition of the TNM Classification for Lung and Pleural Tumors [17]. RFS was defined as the period from the date of surgery to the date of recurrence or any cause of death, wherein patients without recurrence were censored in the last observation period. Adjuvant chemotherapy included patients who completed at least three courses of platinum-based adjuvant chemotherapy or 2 years of oral tegafur-uracil after surgery.

### Pathological examination and EGFR mutation analysis

Pathological diagnosis was made by a pathologist (Y.T.) based on hematoxylin and eosin staining of the tissue sections of formalin-fixed paraffin-embedded specimens. Tumor invasion size was evaluated by Elastica von Gieson (EVG) staining. Blood vessel invasion (BVI) and pleural invasion were also evaluated using EVG staining. Alcian blue-periodic acid Schiff and thyroid transcription factor-1 staining were performed for the supplemental diagnosis of adenocarcinoma, with additional D2–40 staining for lymphatic vessel invasion (LVI). BVI was defined as the destruction of blood vessel walls or invasion of tumor cells into the lumen of blood vessels in HE- or EVG-stained sections of the resected lung specimens. LVI was defined as tumor cell invasion into the lumen of lymphatic vessels in HE- or D2–40-stained sections. Tumor DNA was extracted from formalin-fixed paraffin-embedded surgically resected specimens, and EGFR mutations were analyzed using the Cobas® EGFR mutation test v2 [18, 19], the Cycleave/fragment method [20], and the loop-hybrid mobility shift assay method [21].

Adenocarcinoma subtypes were classified according to the International Association for the Study of Lung Cancer/American Thoracic Society/European Respiratory Society International Multidisciplinary Classification of Lung Adenocarcinoma (2011 IASLC/ATS/ERS classification) [22]. Based on the predominance of the components, lung adenocarcinoma specimens were categorized as invasive lepidic/acinar, papillary/mucinous/solid/micropapillary adenocarcinoma, and others. If lepidic, solid, or micropapillary components were observed in more than 1% of the lung cancer tissue sections, they were defined as lepidic, solid, or micropapillary components (+), respectively. Based on the classification by Yoshizawa et al. [23], adenocarcinoma in situ and minimally invasive adenocarcinoma were defined as low-grade adenocarcinoma, invasive lepidic adenocarcinoma, invasive acinar adenocarcinoma, invasive papillary adenocarcinoma, and invasive mucinous adenocarcinoma as intermediate-grade adenocarcinoma, and invasive solid adenocarcinoma and invasive micropapillary adenocarcinoma as high-grade adenocarcinoma.

### Postoperative surveillance and definitions of recurrence

Patients were routinely examined on an outpatient basis every 3–6 months 1–3 years after resection of lung cancer and every 6–12 months after 4–5 years, with blood tumor markers, radiographs, and CT. If symptoms suggestive of recurrence appeared during follow-up, PET-CT or bone scintigraphy, head magnetic resonance imaging (MRI), or contrast-enhanced CT were added as appropriate. If any relapse was confirmed, we examined further recurrence sites using head MRI, CT, PET-CT, or bone scintigraphy. Based on the results of these examinations, the sites of recurrence and treatment after recurrence were determined at a joint conference consisting of thoracic surgeons, respiratory physicians, pathologists, and radiologists. Intrathoracic recurrence included cervicothoracic lymph node recurrence (mediastinal, hilar, cervical, and supraclavicular lymph node metastases), lung recurrence, and pleural dissemination. On the other hand, distant recurrence included central nerve system (CNS) recurrence (brain metastasis and meningeal dissemination), abdominal organ metastasis recurrence (liver, adrenal gland, intra-abdominal lymph node), and bone recurrence.

### Statistical analysis

In comparison of clinicopathologic background of patients between Mt and Wt, continuous variables were compared using the Mann–Whitney U test, and categorical variables were compared between groups using Fisher’s exact tests. RFS of Mt and Wt patients was analyzed using the Kaplan–Meier method and compared using log-rank tests. Univariable and multivariable analyses were performed using the Cox proportional hazard regression model with the following variables: age (> 65 years), sex, smoking history, laterality, carcinoembryonic antigen (CEA) level (> 10 ng/ml), CT tumor size (> 4.0 cm), consolidation tumor size (> 4.0 cm), presence or absence of ground glass opacity (GGO), PET max standardized uptake value (SUV), surgical procedure, histological grade of adenocarcinoma, BVI, LVI, pleural invasion, lymph node metastasis (pN), predominant component of the adenocarcinoma, presence or absence of lepidic/solid/micropapillary components, adjuvant chemotherapy, and EGFR mutation subtype. Cut-off values for maxSUV was determined using receiver operating characteristic curve analysis. The cumulative incidence of intrathoracic/distant recurrence and recurrence to the CNS was analyzed using Gray’s test. Statistical significance was set at *P* < 0.05.

## Results

The median follow-up period was 38.4 months. Among the 208 Mt, 96 (46.2%) were Ex21 and 112 (53.8%) were Ex19 (Table 1). A smaller CT tumor size and consolidation tumor size, lower frequency of GGO-absent tumors, lower PET maxSUV, higher frequency of invasive micropapillary adenocarcinoma, and lower frequency of invasive solid adenocarcinoma or high-grade adenocarcinoma were observed in Mt than in Wt (Table 1). However, LVI, pN (especially pN2), overall recurrence, and intrathoracic recurrence were observed more frequently in the Mt group than in the Wt group (Table 1).

**Table 1:** Comparison of clinicopathological features between epidermal growth factor receptor mutant and mutation-wild patients with pathological stage IB-IIIA lung cancer

Total *n* = 535	Mt (*n* = 208)	Wt (*n* = 358)	*p* values^a)^
Age	70 (35–90)	70 (36–90)	0.789^b)^
Male, (%)	86 (41.3)	260 (72.6)	< 0.001
Smoking history +, (%)	93 (44.7)	287 (80.2)	< 0.001
Left side, (%)	74 (35.6)	142 (39.7)	0.370
CEA (> 10 ng/ml)	34 (16.3)	61 (17.0)	0.907
CT tumor size, (cm)	3.1 (1.5–9.2)	3.4 (0.8–11.6)	0.033^b)^
Consolidation size, (cm)	2.7 (1.1–9.2)	3.2 (0.6–11.0)	0.001^b)^
GGO absent, (%)	128 (61.5)	306 (85.5)	< 0.001
PET maxSUV	4.9 (0.89–21.2)	7.4 (0–33.7)	< 0.001^b)^
Lobectomy	199 (95.7)	334 (93.3)	0.270
Invasive lepidic adenocarcinoma, (%)	24 (11.5)	24 (6.7)	0.060
Invasive acinar adenocarcinoma, (%)	78 (37.5)	76 (21.2)	< 0.001
Invasive papillary adenocarcinoma, (%)	77 (37.0)	85 (23.7)	0.001
Invasive mucinous adenocarcinoma, (%)	1 (0.5)	44 (12.3)	< 0.001
Invasive solid adenocarcinoma, (%)	14 (6.7)	104 (29.1)	< 0.001
Invasive micropapillary adenocarcinoma, (%)	11 (5.3)	4 (1.1)	0.004
High-grade adenocarcinoma	28 (13.5)	126 (45.2)	< 0.001
Lymphatic vessel invasion +, (%)	78 (37.5)	86 (24.0)	0.002
Blood vessel invasion +, (%)	122 (58.7)	198 (56.3)	0.482
Pleural invasion +, (%)	125 (60.1)	197 (55.3)	0.253
Nodal metastasis +, (%)	107 (51.4)	125 (34.9)	< 0.001
pN1	47 (22.6)	66 (18.4)	0.233
pN2	60 (28.8)	59 (16.5)	< 0.001
Lepidic component +, (%)	185 (88.9)	195 (54.5)	< 0.001
Solid component +, (%)	109 (52.4)	240 (67.0)	< 0.001
Micropapillary component +, (%)	121 (58.2)	128 (35.8)	< 0.001
Adjuvant chemotherapy, (%)	84 (40.4)	124 (34.6)	0.176
Pathological stage			
Stage IB	82 (39.4)	142 (39.7)	
Stage II	54 (26.0)	128 (35.8)	
Stage IIIA	72 (34.6)	88 (24.6)	0.014
Recurrence +, (%)	92 (44.2)	121 (33.8)	0.015
Initial site of recurrence, (%)			
Central nerve system	17 (8.2)	16 (4.5)	0.192
Bone	21 (10.1)	23 (6.4)	0.143
Abdominal organ	10 (4.8)	24 (6.7)	0.463
Lung	29 (13.9)	41 (11.5)	0.428
Cervico-thoracic lymph-node	38 (18.3)	43 (12.0)	0.047
Pleural dissemination	23 (11.1)	15 (4.2)	0.003
Distant or intrathoracic, (%)			
Distant	37 (17.8)	57 (15.9)	0.639
Intrathoracic	76 (36.5)	84 (23.5)	0.001
EGFR mutation, (%)			
Exon 21 L858R	96 (46.2)		
Exon 19 deletion	112 (53.8)		

*CT* Computed tomography, *GGO* Ground glass opacity, *PET* Positron emission tomography, *SUV* Standardized uptake value, *EGFR* Epidermal growth factor receptor, *Mt* EGFR mutant lung cancer, *Wt* EGFR mutation-wild lung cancer

^a^ Fisher’s exact test

^b^ Mann-Whitney U test

**Fig. 1:**
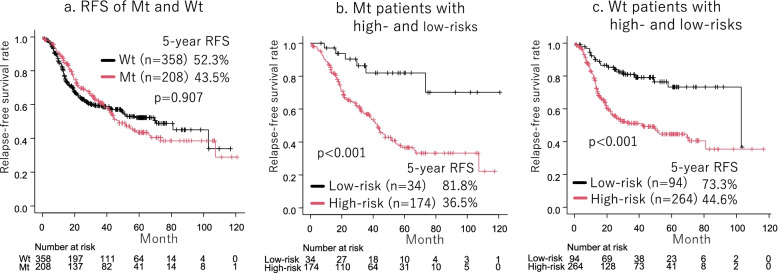
Relapse-free survival (RFS) of Mt and Wt patients did not differ (*p* = 0.907, (**a**)). The RFS of Mt high-risk patient was significantly worse than Mt low-risk patients (36.5% vs. 81.8%, *p* < 0.001, (**b**)). The RFS of Wt high-risk patients was significantly worse than Wt low-risk patients (44.6% vs. 73.3%, *p* < 0.001, (**c**))

The 5-year RFS rates for the Mt and Wt groups were 43.5 and 52.3%, respectively (*p* = 0.907, Fig. 1a). In patients with Mt, smoking history (hazard ratio [HR], 1.49; 95% confidence interval [CI], 1.00–2.22; *p* = 0.049), BVI (HR, 1.84; 95% CI, 1.09–3.12; *p* = 0.023), and pN (HR, 1.96; 95% CI, 1.23–3.12; *p* = 0.005) were poor prognostic factors for RFS (Table 2). Defining Mt patients with smoking history, BVI, or pN as Mt high-risk patients (*n* = 174) and Mt patients with none of the above as Mt low-risk patients (*n* = 34), the RFS of Mt high-risk patients was significantly lower than that of Mt low-risk patients (36.5% vs. 81.8%, *p* < 0.001, Fig. 1b).

**Table 2:** Univariable and multivariable analyses of recurrence-free survival of patients with EGFR-Mt lung cancer

Recurrent-fee survival Variable	Univariable analysis			Multivariable analysis		
	HR	95% CI	*p* value	HR	95% CI	*p* value
Age (≥65)	0.95	0.64–1.44	0.829			
Gender (Male)	1.21	0.82–1.81	0.333			
Smoking history (+)	1.43	0.96–2.12	0.079	1.49	1.00–2.22	0.049
Side (left)	1.48	0.99–2.23	0.058	1.36	0.90–2.06	0.139
CEA (> 10 ng/ml)	1.85	1.14–3.00	0.013	1.29	0.77–2.14	0.334
CT tumor size (> 4.0 cm)	1.31	0.85–2.02	0.224			
Consolidation size (> 4.0 cm)	1.43	0.90–2.27	0.127			
GGO (−)	1.48	0.98–2.24	0.066	1.24	0.80–1.92	0.337
PET maxSUV (≥6.0)	2.21	1.48–3.28	< 0.001	1.37	0.88–2.12	0.161
Surgical procedure (segmentectomy)	0.46	0.11–1.88	0.282			
Invasive lepidic adenocarcinoma	0.61	0.30–1.26	0.182			
Invasive acinar adenocarcinoma	0.78	0.52–1.17	0.225			
Invasive papillary adenocarcinoma	1.29	0.85–1.95	0.225			
Invasive solid adenocarcinoma	1.64	0.83–3.27	0.156			
Invasive micropapillary adenocarcinoma	1.18	0.52–2.71	0.690			
High grade adenocarcinoma	1.54	0.91–2.61	0.104			
Lymphatic vessel invasion (+)	2.02	1.36–3.00	< 0.001	1.25	0.81–1.93	0.308
Blood vessel invasion (+)	2.96	1.86–4.73	< 0.001	1.84	1.09–3.12	0.023
Pleural invasion (+)	1.10	0.73–1.66	0.645			
Nodal metastasis (+)	2.71	1.77–4.14	< 0.001	1.96	1.23–3.12	0.005
Lepidic component (+)	1.05	0.56–1.97	0.877			
Solid component (+)	1.02	0.69–1.52	0.916			
Micropapillary component (+)	1.34	0.89–2.03	0.161			
Adjuvant chemotherapy (%)	1.02	0.69–1.53	0.906			
EGFR exon 21L858R	1.01	0.68–1.50	0.962			

*CT* Computed tomography, *GGO* Ground glass opacity, *PET* Positron emission tomography, *SUV* Standardized uptake value, *EGFR* Epidermal growth factor receptor, *HR* Hazards ratio, *CI* Confidence interval

In Wt patients, PET maxSUV ≥6.0 (HR, 1.53; 95% CI, 1.02–2.31; *p* = 0.042), LVI (HR, 1.54; 95% CI, 1.03–2.30; *p* = 0.036), pN (HR, 1.79; 95% CI, 1.23–2.60; *p* = 0.002) and adjuvant chemotherapy (HR, 0.60; 95% CI, 0.42–0.88; *p* = 0.008) were independent prognostic factors for RFS (Table 3). Defining Wt patients with PET maxSUV ≥6.0, LVI, or pN as Wt high-risk patients (*n* = 264) and Wt patients with none of the above as low-risk patients (*n* = 94), the RFS of Wt high-risk patients was significantly lower than that of Wt low-risk patients (44.6% vs. 73.3%, *p* < 0.001, Fig. 1c).

**Table 3:** Univariable and multivariable analyses of recurrence-free survival of patients with EGFR-Wt lung cancer

Recurrent-fee survival Variable	Univariable analysis			Multivariable analysis		
	HR	95% CI	*p* value	HR	95% CI	*p* value
Age (≥65)	1.28	0.88–1.87	0.189			
Gender (Male)	1.66	1.13–2.47	0.013	1.71	0.97–3.00	0.062
Smoking history (+)	1.46	0.93–2.28	0.097	0.97	0.51–1.83	0.915
Side (left)	0.81	0.58–1.14	0.228			
CEA (> 10 ng/ml)	1.33	0.88–2.00	0.172			
CT tumor size (> 4.0 cm)	1.46	1.05–2.03	0.025	2.07	0.87–4.94	0.102
Consolidation size (> 4.0 cm)	1.47	1.05–2.05	0.025	0.84	0.34–2.07	0.707
GGO (−)	0.90	0.58–1.42	0.665			
PET maxSUV (≥6.0)	1.98	1.38–2.83	< 0.001	1.53	1.02–2.31	0.042
Surgical procedure (segmentectomy)	1.19	0.64–2.21	0.575			
Invasive lepidic adenocarcinoma	0.49	0.22–1.12	0.093	0.71	0.31–1.67	0.438
Invasive acinar adenocarcinoma	1.12	0.76–1.63	0.576			
Invasive papillary adenocarcinoma	1.24	0.86–1.80	0.244			
Invasive mucinous adenocarcinoma	0.58	0.32–1.05	0.072	0.74	0.36–1.49	0.393
Invasive solid adenocarcinoma	1.01	0.70–1.45	0.963			
Invasive micropapillary adenocarcinoma	1.29	0.32–5.21	0.720			
High-grade adenocarcinoma	1.17	0.83–1.64	0.378			
Lymphatic vessel invasion (+)	1.84	1.30–2.61	0.001	1.54	1.03–2.30	0.036
Blood vessel invasion (+)	1.55	1.10–2.17	0.011	1.18	0.79–1.77	0.424
Pleural invasion (+)	1.09	0.78–1.51	0.625			
Nodal metastasis (+)	2.90	1.94–4.34	< 0.001	1.79	1.23–2.60	0.002
Lepidic component (+)	1.30	0.94–1.80	0.118			
Solid component (+)	1.49	1.03–2.16	0.035	1.00	0.63–1.56	0.987
Micropapillary component (+)	1.35	0.97–1.87	0.078	1.21	0.85–1.73	0.298
Adjuvant chemotherapy (+)	0.73	0.51–1.04	0.081	0.60	0.42–0.88	0.008

*CT* Computed tomography, *GGO* Ground glass opacity, *PET* Positron emission tomography, *SUV* Standardized uptake value, *EGFR* Epidermal growth factor receptor, *HR* Hazards ratio, *CI* Confidence interval

**Fig. 2:**
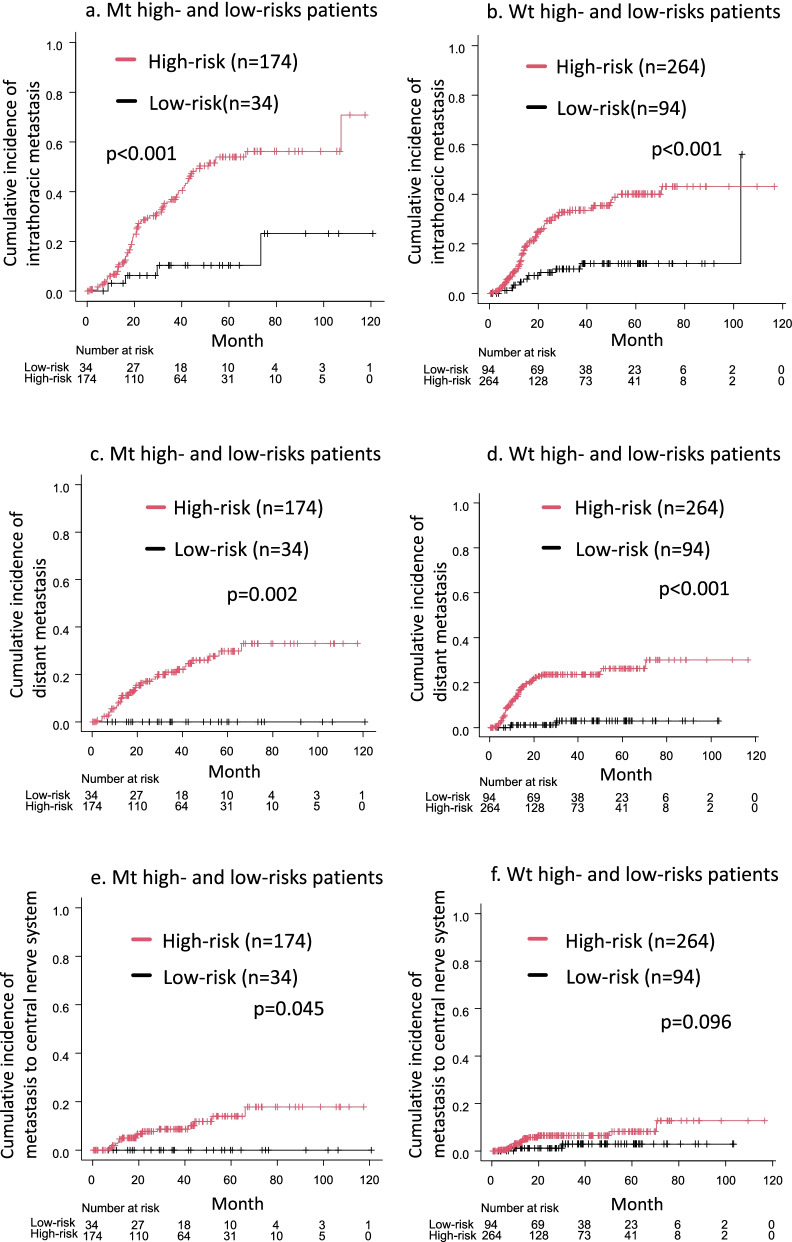
The cumulative incidence of intrathoracic metastasis, distant metastasis, and metastasis to the central nervous system (CNS) was significantly higher in Mt high-risk patients than in Mt low-risk patients (**a**, **c**, **e**). The cumulative incidence of intrathoracic and distant metastases was higher in Wt high-risk patients than in Wt low-risk patients (**b**, **d**). The cumulative incidence of CNS metastasis tended to be higher in Wt high-risk patients than in Wt low-risk patients (**f**)

The cumulative incidence of intrathoracic metastasis (*p* < 0.001, Fig. 2a), distant metastasis (*p* = 0.002, Fig. 2c), and CNS metastasis (*p* = 0.045, Fig. 2e) was significantly higher in Mt high-risk patients relative to Mt low-risk patients. The cumulative incidence of intrathoracic metastasis (*p* < 0.001, Fig. 2b) and distant metastasis (*p* < 0.001, Fig. 2d) was significantly higher in Wt high-risk patients than in Wt low-risk patients, and the cumulative incidence of CNS metastasis tended to be higher in Wt-high-risk patients than in Wt-low-risk patients (*p* = 0.096, Fig. 2f).

**Fig. 3:**
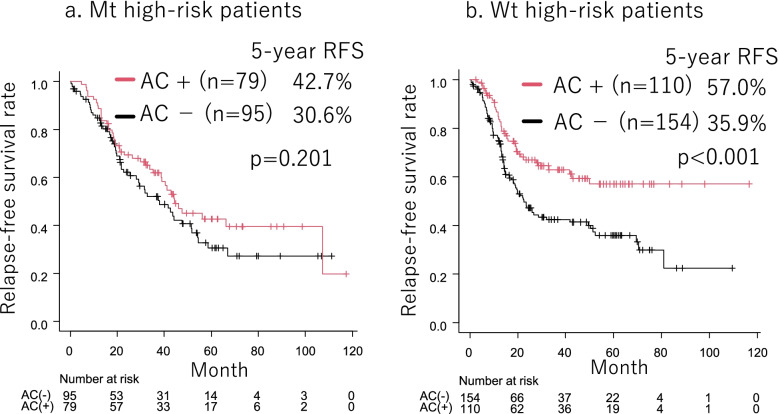
There was no difference in RFS between patients treated with or without adjuvant chemotherapy in Mt high-risk patients (*p* = 0.201, (**a**)). In Wt high-risk patients, RFS was significantly better in patients who received adjuvant chemotherapy than in those who did not receive adjuvant chemotherapy (*p* < 0.001, (**b**))

Figure 3 shows the difference in RFS between patients treated with and without adjuvant chemotherapy in the Mt and Wt high-risk groups. There was no difference in RFS between patients treated with or without adjuvant chemotherapy in Mt high-risk patients (*p* = 0.201, Fig. 3a). In Wt high-risk patients, RFS was significantly better in patients who received adjuvant chemotherapy than in those who did not receive adjuvant chemotherapy (*p* < 0.001, Fig. 3b).

## Discussion

This is the first study to examine prognostic factors for RFS after complete resection of stage IB-IIIA (8th edition TNM classification) pulmonary adenocarcinoma according to EGFR mutation status. Smoking history, BVI, and pN were prognostic factors for RFS of Mt patients; however, adjuvant chemotherapy did not contribute to RFS in Mt patients. In contrast, PET maxSUV≥6.0, LVI, pN, and adjuvant chemotherapy were prognostic factors for RFS in Wt patients. Mt and Wt high-risk patients had a higher cumulative incidence of recurrence than Mt and Wt low-risk patients, respectively.

In this study, the RFS of patients with stage IB-IIIA Mt was 43.5%. This reflects a poor outcome, and there is room for prognostic improvement. In the present study, there was no statistically significant difference in the RFS between Mt and Wt. This was due to the crossover of RFS curves at approximately 40 months after surgery. In our previous study, we showed that Wt recurrence occurred early postoperatively, whereas Mt recurrence tended to be slower than that of Wt [24]. The reason for the crossover may be related to Mt recurrence later in the postoperative period, although recurrence occurred more frequently than in Wt. Conversely, recurrence and smoking-related non-lung cancer deaths in Wt occurred earlier in the postoperative period. There is an urgent need for evidence on adjuvant therapy to reduce recurrence and improve the prognosis of patients with stage IB-IIIA Mt.

Currently, adjuvant EGFR-TKIs may prolong RFS in patients with Mt, but not OS. In RADIENT trial, adjuvant erlotinib therapy prolonged RFS with compared with placebo after complete resection of stage IB-IIIA Mt (46.4 months vs. 28.5 months, *p* = 0.039) but not OS [11]. In ADJUVANT trial, adjuvant gefitinib therapy pathologic stage II-IIIA Mt prolonged RFS compare with platinum-based combination therapy (28.7 months vs. 18.0 months, HR 0.60, *p* = 0.005) [12], however, no difference was observed in OS between the two therapies [13]. In the IMPACT study, adjuvant gefitinib therapy for stage II-IIIA Mt did not prolong RFS (Median RFS 35.9 months vs. 25.1 months, *p* = 0.63) and OS (5 years OS 78.0% vs. 74.6%, *p* = 0.89) compared with platinum-based adjuvant chemotherapy [10]. The most recent ADAURA trial showed significantly better RFS with adjuvant osimertinib therapy compared with placebo for pathologic stage IB-IIIA Mt (not reached vs 27.5 months, HR 0.20, *p* < 0.001) [14]. In the ADAURA trial, the incidence of central nervous system (CNS) events was 2% in the osimertinib group and 11% in the placebo group, and CNS-RFS was prolonged with adjuvant osimertinib therapy [14]. However, OS results have not yet been reported.

Various adverse events were reported in these trials. Grade ≥ 3 and grade ≥ 4 adverse events of adjuvant EGFR-TKIs were reported to be 12–41.7% and 1–4%, respectively [10, 12, 14]. Therefore, adjuvant EGFR-TKIs should be administered only to patients with poor RFS after curative surgery. However, few studies have examined the prognostic factors for RFS by EGFR mutation status in stage IB-IIIA adenocarcinoma patients whose stages are usually indicated for adjuvant therapy. Ni et al. reported that among stage I-III Mt patients (*n* = 531), tumor size, N stage, Ki67, and CK20 were risk factors for overall recurrence [25]. Saw et al. reported that higher stage, nonacinar and nonlepidic adenocarcinoma subtype, sublobar resection, positive resection margins, and lymphovascular invasion (LI) were independent risk factors for recurrence in stage IA-IIIA Mt (*n* = 389), while higher stage and LI were independent risk factors for recurrence in stage IA-IIIA Wt (*n* = 334) [26]. The present study revealed that smoking history, BVI, and pN were poor prognostic factors for RFS among patients with stage IB-IIIA Mt. Although pN was a common poor prognostic factor for RFS in Wt and Mt patients, other risk factors differed. This study suggests that it is important to recognize that clinicopathological poor prognostic factors of RFS for stage IB-IIIA adenocarcinoma vary according to EGFR mutation status.

LI combining BVI and LVI has been reported to be associated with distant recurrence and early recurrence after complete resection of NSCLC [27–29], and is a poor prognostic factor for RFS [30, 31] and OS [32]. LVI alone increases the risk of recurrence. Harada et al. reported that LVI was a poor prognostic factor for OS after complete resection of stage I NSCLC, whereas BVI was not [30]. In contrast, a meta-analysis reported that BVI was a poor prognostic factor for RFS after resection of stage I NSCLC [31]. Kato et al. reported that LVI and BVI were risk factors for recurrence [33]. Mimae et al. reported that LVI and BVI were independent poor prognostic factors for RFS among pN (−) lung adenocarcinoma patients, whereas only BVI was a risk factor for recurrence in patients with pN (+) lung adenocarcinoma [34]. None of these studies have examined the association between EGFR mutations. The present study revealed that BVI is a poor prognostic factor for RFS in patients with Mt, and LVI is a poor prognostic factor for RFS in patients with Wt. It is suggested that LI should be analyzed separately for each EGFR mutation status in LVI and BVI.

The reason why BVI is a prognostic factor for RFS is unclear. In tumor cells with high EGFR expression, tumor cells produce vascular endothelial growth factor (VEGF) and interleukin-8, which regulate and promote intratumoral vascular invasion [35]. Moreover, EGFR signaling induces angiogenic factors from mesenchymal stem cells in tumors and regulates tumor cell migration [36]. C-type lectin 11A, which promotes vascular endothelial cell differentiation, is highly expressed in EGFR-mutant lung cancer cells and promotes angiogenesis triggered by VEGF [37]. These basic research findings showed the possibility that Mt may metastasize more frequently by efficient angiogenesis than Wt. Three angiogenesis inhibitors are currently used for advanced primary lung cancer: bevacizumab, ramucirumab, and nintedanib. Because Mt patients with BVI are at a high risk of recurrence, future clinical trials of adjuvant therapy with these angiogenesis inhibitors in addition to EGFR-TKIs may be considered.

In both Mt and Wt, pN was an important prognostic factor, and this result was consistent with a previous report [27, 28, 34]. In the present study, smoking history was also a prognostic factor in Mt smoking and has been reported to have a shorter OS than non-smokers in unresectable stage IIIB-IV Mt, and the prognosis is worsened by the increased amount of smoking [38]. Progression-free survival after EGFR-TKI treatment for unresectable advanced Mt has been reported to be shorter in smokers than in non-smokers [39]. Because lung cancer in smoking patients had a 10-fold higher mutation burden than those in non-smokers [40, 41], it is possible that Mt in patients with a smoking history had higher cell proliferative activity than those without a smoking history. Further molecular pathological analysis may be needed for Mt patients with a history of smoking.

It has been reported that solid predominant adenocarcinoma has a higher maxSUV than other histologic types of lung adenocarcinoma [42]. In this study, a lower solid component rate, higher lepidic component rate, and lower frequency of high-grade adenocarcinoma in Mt than in Wt resulted in a lower maxSUV in Mt than in Wt. This result was consistent with previous studies showing lower PET maxSUVs in Mt than in Wt [2, 26]. In the present study, PET maxSUV≥6.0 was a poor prognostic factor for RFS in Wt, but not in Mt. Because of the low potential of 18F-fluorodeoxyglucose (FDG) accumulation in Mt, maxSUV may not accurately predict the prognosis of patients with Mt. In contrast, Wt has a high potential for FDG accumulation and is useful in predicting prognosis after resection of stage IB-IIIA Wt.

The efficacy of adjuvant chemotherapy for Mt has been reported to be lower than that for Wt in stage I [43] and stage II/III [44] lung adenocarcinoma in a propensity-matched analysis. Consistent with previous reports, the present study showed that adjuvant chemotherapy did not affect RFS in stage IB-IIIA Mt and Mt high-risk patients; therefore, adjuvant chemotherapy may not be necessary for Mt. Mt patients who had a smoking history, BVI, and pN were more likely to relapse. These high-risk Mt patients are candidates for adjuvant therapy other than chemotherapy, such as EGFR-TKI, and further clinical trials are warranted. Surprisingly, Mt patients with none of these factors, that is, Mt low-risk patients, did not develop distant metastasis (Fig. 2a, c, e), suggesting that adjuvant therapy may not be necessary for Mt low-risk patients. In contrast, adjuvant chemotherapy was a favor prognostic factor for RFS in patients with Wt. Because adjuvant chemotherapy may improve the prognosis of Wt patients with PET SUVmax ≥6.0, LVI, or pN, that is, Wt high-risk patients, should receive aggressive adjuvant chemotherapy.

Our study has several limitations. First, this was a single-center, retrospective study. Second, among the 703 patients with pathological stage IB-IIIA primary lung adenocarcinoma during the analysis period, 8.3% and 8.5% of the patients did not undergo EGFR mutation analysis and PET-CT, respectively. These patients were excluded from this study, which may have caused a selection bias. Third, Wt patients are a heterogeneous population that includes patients with KRAS, ALK, and ROS-1 mutation lung cancer. It is necessary to examine the poor prognostic factors for RFS for each gene mutation in lung cancer in the future.

## Conclusions

In conclusion, we found that poor prognostic factors for RFS in stage IB-IIIA differ according to EGFR mutation status. Smoking history, BVI, and pN were unfavorable factors for RFS in Mt patients. In contrast, PET maxSUV≥6.0, LVI, pN, and adjuvant chemotherapy were prognostic factors for RFS in Wt patient.

## Data Availability

The datasets used and/or analyzed during the current study are available from corresponding author on reasonable request.

## Abbreviations

BVI: Blood vessel invasion
CEA: Carcinoembryonic antigen
CI: Confidence interval
CNS: Central nerve system
CT: Computed tomography
EGFR: Epidermal growth factor receptor
Ex19: EGFR exon 19 deletion
Ex21: EGFR exon 21 L858R point mutation
FDG: Fluorodeoxyglucose
GGO: Ground glass opacity
HR: Hazard ratio
LI: Lymphovascular invasion
LVI: Lymphatic vessel invasion
MRI: Magnetic resonance imaging
Mt: EGFR mutation lung cancer
NSCLC: Non-small cell lung cancers
OS: Overall survival
PET: Positron emission tomography
pN: Lymph node metastasis
RFS: Relapse-free survival
SUV: Standardized uptake value
TKI: Tyrosine kinase inhibitors
VEGF: Vascular endothelial growth factor
Wt: EGFR mutation wild-type lung cancer

## References

[1] Rosell R, Moran T, Queralt C, Porta R, Cardenal F, Camps C, Majem M, Lopez-Vivanco G, Isla D, Provencio M, Insa A, Massuti B, Gonzalez-Larriba JL, Paz-Ares L, Bover I, Garcia-Campelo R, Moreno MA, Catot S, Rolfo C, Reguart N, Palmero R, Sánchez JM, Bastus R, Mayo C, Bertran-Alamillo J, Molina MA, Sanchez JJ, Taron M, Spanish Lung Cancer Group (2009). Screening for epidermal growth factor receptor mutations in lung cancer. N Engl J Med.

[2] Isaka T, Yokose T, Ito H, Nagata M, Furumoto H, Nishii T, Katayama K, Yamada K, Nakayama H, Masuda M (2015). Correlations between the EGFR mutation status and Clinicopathological features of clinical stage I lung adenocarcinoma. Medicine (Baltimore).

[3] Weinstein IB (2002). Cancer. Addiction to oncogenes--the Achilles heal of cancer. Science..

[4] Ramalingam SS, Vansteenkiste J, Planchard D, Cho BC, Gray JE, Ohe Y, Zhou C, Reungwetwattana T, Cheng Y, Chewaskulyong B, Shah R, Cobo M, Lee KH, Cheema P, Tiseo M, John T, Lin MC, Imamura F, Kurata T, Todd A, Hodge R, Saggese M, Rukazenkov Y, Soria JC, FLAURA Investigators (2020). Overall survival with Osimertinib in untreated, EGFR-mutated advanced NSCLC. N Engl J Med.

[5] Wu YL, Zhou C, Hu CP, Feng J, Lu S, Huang Y, Li W, Hou M, Shi JH, Lee KY, Xu CR, Massey D, Kim M, Shi Y, Geater SL (2014). Afatinib versus cisplatin plus gemcitabine for first-line treatment of Asian patients with advanced non-small-cell lung cancer harbouring EGFR mutations (LUX-lung 6): an open-label, randomised phase 3 trial. Lancet Oncol.

[6] Hosomi Y, Morita S, Sugawara S, Kato T, Fukuhara T, Gemma A, Takahashi K, Fujita Y, Harada T, Minato K, Takamura K, Hagiwara K, Kobayashi K, Nukiwa T, Inoue A, North-East Japan Study Group (2020). Gefitinib alone versus Gefitinib plus chemotherapy for non-small-cell lung Cancer with mutated epidermal growth factor receptor: NEJ009 study. J Clin Oncol.

[7] Kerr KM, Nicolson MC (2013). Prognostic factors in resected lung carcinomas. EJC Suppl.

[8] Isaka T, Ito H, Nakayama H, Yokose T, Yamada K, Masuda M (2020). Effect of epidermal growth factor receptor mutation on early-stage non-small cell lung cancer according to the 8th TNM classification. Lung Cancer.

[9] Isaka T, Ito H, Nakayama H, Yokose T, Saito H, Adachi H, Miura J, Shigefuku S, Kikuchi A, Rino Y (2021). Effect of epidermal growth factor receptor gene mutation on the prognosis of pathological stage II-IIIA (8th edition TNM classification) primary lung cancer after curative surgery. Lung Cancer.

[10] Tada H, Mitsudomi T, Misumi T, Sugio K, Tsuboi M, Okamoto I, Iwamoto Y, Sakakura N, Sugawara S, Atagi S, Takahashi T, Hayashi H, Okada M, Inokawa H, Yoshioka H, Takahashi K, Higashiyama M, Yoshino I, Nakagawa K, West Japan Oncology Group (2022). Randomized phase III study of Gefitinib versus cisplatin plus Vinorelbine for patients with resected stage II-IIIA non-small-cell lung Cancer with EGFR mutation (IMPACT). J Clin Oncol.

[11] Kelly K, Altorki NK, Eberhardt WE, O'Brien ME, Spigel DR, Crinò L, Tsai CM, Kim JH, Cho EK, Hoffman PC, Orlov SV, Serwatowski P, Wang J, Foley MA, Horan JD, Shepherd FA (2015). Adjuvant Erlotinib versus placebo in patients with stage IB-IIIA non-small-cell lung Cancer (RADIANT): a randomized, double-blind, phase III trial. J Clin Oncol.

[12] Zhong WZ, Wang Q, Mao WM, Xu ST, Wu L, Shen Y, Liu YY, Chen C, Cheng Y, Xu L, Wang J, Fei K, Li XF, Li J, Huang C, Liu ZD, Xu S, Chen KN, Xu SD, Liu LX, Yu P, Wang BH, Ma HT, Yan HH, Yang XN, Zhou Q, Wu YL, ADJUVANT investigators. (2018). Gefitinib versus vinorelbine plus cisplatin as adjuvant treatment for stage II-IIIA (N1-N2) EGFR-mutant NSCLC (ADJUVANT/CTONG1104): a randomised, open-label, phase 3 study. Lancet Oncol..

[13] Zhong WZ, Wang Q, Mao WM, Xu ST, Wu L, Wei YC, Liu YY, Chen C, Cheng Y, Yin R, Yang F, Ren SX, Li XF, Li J, Huang C, Liu ZD, Xu S, Chen KN, Xu SD, Liu LX, Yu P, Wang BH, Ma HT, Yang JJ, Yan HH, Yang XN, Liu SY, Zhou Q, Wu YL (2021). Gefitinib versus Vinorelbine plus cisplatin as adjuvant treatment for stage II-IIIA (N1-N2) EGFR-mutant NSCLC: final overall survival analysis of CTONG1104 phase III trial. J Clin Oncol.

[14] Wu YL, Tsuboi M, He J, John T, Grohe C, Majem M, Goldman JW, Laktionov K, Kim SW, Kato T, Vu HV, Lu S, Lee KY, Akewanlop C, Yu CJ, de Marinis F, Bonanno L, Domine M, Shepherd FA, Zeng L, Hodge R, Atasoy A, Rukazenkov Y, Herbst RS, ADAURA Investigators (2020). Osimertinib in resected EGFR-mutated non-small-cell lung Cancer. N Engl J Med.

[15] D'Antonio C, Passaro A, Gori B, Del Signore E, Migliorino MR, Ricciardi S, Fulvi A, de Marinis F (2014). Bone and brain metastasis in lung cancer: recent advances in therapeutic strategies. Ther Adv Med Oncol.

[16] Isaka T, Ito H, Nakayama H, Yokose T, Saito H, Masuda M (2022). Impact of the initial site of recurrence on prognosis after curative surgery for primary lung cancer. Eur J Cardiothorac Surg.

[17] Goldstraw P, Chansky K, Crowley J, Rami-Porta R, Asamura H, Eberhardt WE, Nicholson AG, Groome P, Mitchell A, Bluejack V, International Association for the Study of Lung Cancer Staging and Prognostic Factors Committee, Advisory Boards, and Participating Institutions; International Association for the Study of Lung Cancer Staging and Prognostic Factors Committee Advisory Boards and Participating Institutions (2016). The IASLC lung Cancer staging project: proposals for revision of the TNM stage groupings in the forthcoming (eighth) edition of the TNM classification for lung Cancer. J Thorac Oncol.

[18] Malapelle U, Sirera R, Jantus-Lewintre E, Reclusa P, Calabuig-Fariñas S, Blasco A, Pisapia P, Rolfo C, Camps C (2017). Profile of the Roche cobas® EGFR mutation test v2 for non-small cell lung cancer. Expert Rev Mol Diagn.

[19] Lopez-Rios F, Angulo B, Gomez B, Mair D, Martinez R, Conde E, Shieh F, Tsai J, Vaks J, Current R, Lawrence HJ, Gonzalez de Castro D (2013). Comparison of molecular testing methods for the detection of EGFR mutations in formalin-fixed paraffin-embedded tissue specimens of non-small cell lung cancer. J Clin Pathol.

[20] Yoshida K, Yatabe Y, Park JY (2007). Prospective validation for prediction of gefitinib sensitivity by epidermal growth factor receptor gene mutation in patients with non-small cell lung cancer. J Thorac Oncol.

[21] Matsukuma S, Yoshihara M, Kasai F (2006). Rapid and simple detection of hot spot point mutations of epidermal growth factor receptor, BRAF, and NRAS in cancers using the loophybrid mobility shift assay. J Mol Diagn.

[22] Travis WD, Brambilla E, Burke AP, Marx A, Nicholson AG (2015). WHO classification of Tumours of the lung, pleura, Thymus and heart.

[23] Yoshizawa A, Sumiyoshi S, Sonobe M, Kobayashi M, Fujimoto M, Kawakami F, Tsuruyama T, Travis WD, Date H, Haga H (2013). Validation of the IASLC/ATS/ERS lung adenocarcinoma classification for prognosis and association with EGFR and KRAS gene mutations: analysis of 440 Japanese patients. J Thorac Oncol.

[24] Isaka T, Nakayama H, Ito H, Yokose T, Yamada K, Masuda M. Impact of the epidermal growth factor receptor mutation status on the prognosis of recurrent adenocarcinoma of the lung after curative surgery. BMC Cancer. 2018;18(1):959. 10.1186/s12885-018-4849-9 PMC6173892 30290774

[25] Ni J, Guo T, Li Y, Yang X, Li Y, Zou L, Chu L, Chu X, Li S, Ye L, Zhang Y, Zhu Z (2019). Patterns and risks of postoperative recurrence in completely resected EGFR-mutant non-small cell lung cancer: prognostic significance of routine immunohistochemical markers. Transl Lung Cancer Res.

[26] Saw SPL, Zhou S, Chen J, Lai G, Ang MK, Chua K, Kanesvaran R, Ng QS, Jain A, Tan WL, Rajasekaran T, Lim DWT, Tan A, Fong KW, Takano A, Cheng XM, Lim KH, Koh T, Ong BH, Tan EH, Toh CK, Skanderup AJ, Tan SH, Tan DSW (2021). Association of Clinicopathologic and Molecular Tumor Features with Recurrence in resected early-stage epidermal growth factor receptor-positive non-small cell lung Cancer. JAMA Netw Open.

[27] Park C, Lee IJ, Jang SH, Lee JW (2014). Factors affecting tumor recurrence after curative surgery for NSCLC: impacts of lymphovascular invasion on early tumor recurrence. J Thorac Dis.

[28] Higgins KA, Chino JP, Ready N (2012). Lymphovascular invasion in non-small-cell lung cancer: implications for staging and adjuvant therapy. J Thorac Oncol.

[29] Shiono S, Kanauchi N, Yanagawa N (2014). Stage II-IV lung cancer cases with lymphovascular invasion relapse within 2 years after surgery. Gen Thorac Cardiovasc Surg.

[30] Harada M, Hato T, Horio H (2011). Intratumoral lymphatic vessel involvement is an invasive indicator of completely resected pathologic stage I non-small cell lung cancer. J Thorac Oncol.

[31] Wang J, Chen J, Chen X, Wang B, Li K, Bi J (2011). Blood vessel invasion as a strong independent prognostic indicator in non-small cell lung cancer: a systematic review and meta-analysis. PLoS One.

[32] Wang S, Xu J, Wang R, Qian F, Yang W, Qiao R, Zhang B, Qian J, Yu K, Han B (2018). Adjuvant chemotherapy may improve prognosis after resection of stage I lung cancer with lymphovascular invasion. J Thorac Cardiovasc Surg.

[33] Kato T, Ishikawa K, Aragaki M, Sato M, Okamoto K, Ishibashi T, Kaji M (2012). Angiolymphatic invasion exerts a strong impact on surgical outcomes for stage I lung adenocarcinoma, but not non-adenocarcinoma. Lung Cancer.

[34] Mimae T, Tsutani Y, Miyata Y, Yoshiya T, Ibuki Y, Kushitani K, Takeshima Y, Nakayama H, Okumura S, Yoshimura M, Okada M (2014). Role of lymphatic invasion in the prognosis of patients with clinical node-negative and pathologic node-positive lung adenocarcinoma. J Thorac Cardiovasc Surg.

[35] Minder P, Zajac E, Quigley JP, Deryugina EI (2015). EGFR regulates the development and microarchitecture of intratumoral angiogenic vasculature capable of sustaining cancer cell intravasation. Neoplasia..

[36] De Luca A, Gallo M, Aldinucci D, Ribatti D, Lamura L, D’Alessio A, De Filippi R, Pinto A, Normanno N (2011). Role of the EGFR ligand/receptor system in the secretion of angiogenic factors in mesenchymal stem cells. J Cell Physiol.

[37] Lin TY, Yang CH, Chou HC, Cheng CM, Liu YW, Wang JY, Huang LR, Tsai SF, Huang SF, Chen YR (2022). EGFR mutation-harboring lung Cancer cells produce CLEC11A with endothelial trophic and tumor-promoting activities. Cancers (Basel).

[38] Tseng CH, Chiang CJ, Tseng JS, Yang TY, Hsu KH, Chen KC, Wang CL, Chen CY, Yen SH, Tsai CM, Huang MS, Ho CC, Yu CJ, Tsai YH, Chen JS, Chou TY, Tsai MH, Chen HY, Su KY, Chen JJW, Chen HW, Yu SL, Liu TW, Chang GC (2017). EGFR mutation, smoking, and gender in advanced lung adenocarcinoma. Oncotarget..

[39] Hasegawa Y, Ando M, Maemondo M, Yamamoto S, Isa S, Saka H, Kubo A, Kawaguchi T, Takada M, Rosell R, Kurata T, Ou SH (2015). The role of smoking status on the progression-free survival of non-small cell lung cancer patients harboring activating epidermal growth factor receptor (EGFR) mutations receiving first-line EGFR tyrosine kinase inhibitor versus platinum doublet chemotherapy: a meta-analysis of prospective randomized trials. Oncologist..

[40] Imielinski M, Berger AH, Hammerman PS, Hernandez B, Pugh TJ, Hodis E, Cho J, Suh J, Capelletti M, Sivachenko A, Sougnez C, Auclair D, Lawrence MS, Stojanov P, Cibulskis K, Choi K, de Waal L, Sharifnia T, Brooks A, Greulich H, Banerji S, Zander T, Seidel D, Leenders F, Ansén S, Ludwig C, Engel-Riedel W, Stoelben E, Wolf J, Goparju C, Thompson K, Winckler W, Kwiatkowski D, Johnson BE, Jänne PA, Miller VA, Pao W, Travis WD, Pass HI, Gabriel SB, Lander ES, Thomas RK, Garraway LA, Getz G, Meyerson M (2012). Mapping the hallmarks of lung adenocarcinoma with massively parallel sequencing. Cell..

[41] Govindan R, Ding L, Griffith M, Subramanian J, Dees ND, Kanchi KL, Maher CA, Fulton R, Fulton L, Wallis J, Chen K, Walker J, McDonald S, Bose R, Ornitz D, Xiong D, You M, Dooling DJ, Watson M, Mardis ER, Wilson RK (2012). Genomic landscape of non-small cell lung cancer in smokers and never-smokers. Cell..

[42] Suárez-Piñera M, Belda-Sanchis J, Taus A, Sánchez-Font A, Mestre-Fusco A, Jiménez M, Pijuan L (2018). FDG PET-CT SUVmax and IASLC/ATS/ERS histologic classification: a new profile of lung adenocarcinoma with prognostic value. Am J Nucl Med Mol Imaging.

[43] Tsutani Y, Ito M, Shimada Y, Ito H, Ikeda N, Nakayama H, Okada M (2022). The impact of epidermal growth factor receptor mutation status on adjuvant chemotherapy for patients with high-risk stage I lung adenocarcinoma. J Thorac Cardiovasc Surg.

[44] Isaka T, Ito H, Nakayama H, Yokose T, Katayama K, Yamada K, et al. Efficacy of platinum-based adjuvant chemotherapy on prognosis of pathological stage II/III lung adenocarcinoma based on EGFR mutation status: a propensity score matching analysis. Mol Diagn Ther. 2019;23(5):657–65. 10.1007/s40291-019-00419-9 31347029

